# A novel combination treatment against melanoma with NRAS mutation and therapy resistance

**DOI:** 10.15252/emmm.201708573

**Published:** 2018-04-16

**Authors:** Yanlin Yu

**Affiliations:** ^1^ Laboratory of Cancer Biology and Genetics Center for Cancer Research National Cancer Institutes National Institutes of Health Bethesda MD USA

**Keywords:** Cancer, Pharmacology & Drug Discovery, Skin

## Abstract

Targeted cancer therapies have shown some progress in treating BRAF‐mutant melanoma, but not against NRAS‐mutant and treatment‐resistant melanoma. In this issue of *EMBO Molecular Medicine*, Echevarría‐Vargas *et al* (2018) report that cotargeting BET and MEK pathways efficiently kills immune therapy‐resistant and NRAS‐mutant melanoma tumor cells.

Melanoma is the deadliest form of skin cancer, and its incidence is increasing. More than 50% of melanomas are characterized by mutations in the BRAF gene, which constitutively activates the downstream kinase MEK/MAPK and promotes melanogenesis. The second most prevalent oncogenic mutation in 20–25% of melanomas affects the NRAS gene (Thomas *et al*, 2015). This alters GTP hydrolysis through interfering with the gamma‐phosphate of GTP and causes constitutive activation of the MEK/MAPK as well as activation of PI3K and RAS‐like protein GEFs signaling pathways, which makes NRAS‐mutant melanomas more aggressive and more likely to develop resistance against treatment (Downward, 2015; Fig 1A). Current therapies are not very efficient to treat NRAS‐mutant melanoma owing to its aggressive nature and the complex changes in molecular signaling (Johnson & Puzanov, 2015). Although immunotherapies may offer some hope for patients, these are not mutation‐specific and carry the risk of severe toxicity; in addition, some patients’ melanoma cells developed resistance (Gide *et al*, 2018). Novel strategies and new druggable downstream targets of NRAS are therefore needed to improve the precision and efficiency of anti‐NRAS‐mutant melanoma therapies. The report by Echevarría‐Vargas and colleagues in this issue of *EMBO Molecular Medicine* (Echevarría‐Vargas *et al*, 2018) describes a potential new treatment strategy by targeting BRD4 and TCF19 downstream of NRAS, demonstrating that inhibition of both the BRD4 and MEK pathways arrests and kills both NRAS‐mutant melanoma and immunotherapy‐resistant melanoma cells *in vivo* (Fig 1).

**Figure 1:**
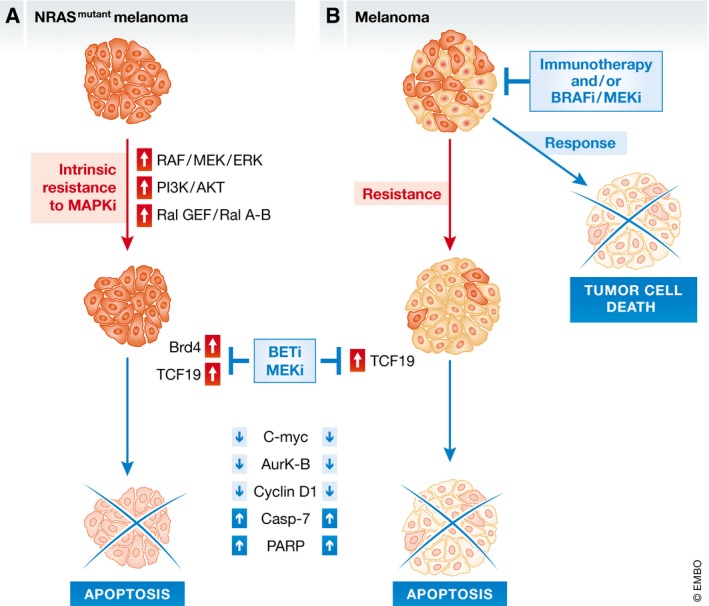
Model of the BET and MEK inhibitor combination therapy to treat melanoma with NRAS mutation and immune therapy resistance (A) NRAS mutation constitutively activates RAF/MEK/ERK, PI3K/AKT, and Ral GEF/Ral A‐B signaling pathways and leads to BRD4 and TCF19 upregulation. The combination therapy of BET and MEK inhibitors reduces BRD4 and TCF19 expressions, which causes apoptosis of tumor cells. (B) Treatment with BRAF/MEK inhibitors or immune therapy targeting immune checkpoints (anti‐PD1 and anti‐CTLA4) shows excellent promises in melanoma only for a few cases, while melanomas with elevated TCF19 protein resist the treatment. A combined therapy of BET and MEK inhibitors causes cell cycle arrest and apoptosis through inhibition of TCF19.

Using the TCGA skin cutaneous melanoma dataset and biopsies from 54 patients to search for new targets to treat NRAS‐mutant melanoma, the authors discovered that BRD4 expression was significantly elevated in tumor cells and correlated with poor survival. An earlier publication by Segura *et al* (2013) had reported that BRD4 is overexpressed in melanomas and that its inhibition blocked cell growth *in vitro* and *in vivo*. BRD4 belongs to the bromodomain and extraterminal domain (BET) family of proteins that “read” histone acetylation and mediates regulation of the cell cycle; as such, BRD4 mutants are involved in tumorigenesis (Shi & Vakoc, 2014). Echevarría‐Vargas and colleagues then knocked down BRD4 by shRNA, which inhibited proliferation of NRAS‐mutant cells. Following this lead, they tested JQ1, a BET inhibitor and anticancer drug, in NRAS‐mutant melanoma cells and found that it reduces their viability—the degree of reduction correlated with the protein levels of BRD4 reversely.

However, JQ1 alone was not enough to block tumor growth in a mouse model (Echevarría‐Vargas *et al*, 2018): As NRAS mutations cause multiple changes in intracellular signaling, targeting only one dysregulated signaling pathway may not be sufficient for lasting therapeutic effects. The authors therefore combined BRD4 inhibition with targeting other deregulated signaling molecules in NRAS‐mutant melanoma cells, such as MEK, PI3K, and CDK4/6 (Fig 1A). They observed that a combination of JQ1 and MEK inhibitors induced cytostasis and triggered apoptosis in tumor cells with obvious implications for clinical use of this combination therapy to treat NRAS‐mutant melanoma with higher levels of BRD4 that are resistant to MAPK inhibitors (Echevarría‐Vargas *et al*, 2018).

In addition, the authors showed by RNA sequencing that the combination of JQ1 and MEK inhibitor significantly repressed three transcriptional regulators: TCF19, E2F1, and E2F3. The latter two are known to play a crucial role in cell cycle control, but the role of TCF19 remains less clear. Through proteomic analysis, Echevarría‐Vargas and colleagues demonstrated that the combination therapy also decreased the level of polo‐like kinase 1 and tumor suppressor retinoblastoma protein RB and increased the amount of CDK inhibitor p27, all of which play a role in regulating the cell cycle. Proteomic analysis further showed activation of the apoptotic caspase‐7, demonstrating that the combination of JQ1 and MEK inhibitor halted the cell cycle and triggered apoptosis (Fig 1A).

Notably, Echevarría‐Vargas *et al* (2018) showed that the combination of BET/MEK inhibitors halted tumor growth of both BRAF/MAPK inhibitor‐resistant and immune therapy‐resistant melanoma cells *in vivo* and that this correlated with reduced levels of TCF19 protein (Fig 1B). Further investigation of the role of TCF19 in activating apoptotic pathways confirmed that TCF19 levels positively correlated with BRD4 protein levels and sensitivity to BET inhibitors; depletion of TCF19 activated the cleavage of caspase‐7 and PARP, and promoted apoptosis of NRAS‐mutant melanoma cells; while the level of TCF19 inversely associated with patient's survival in the cutaneous melanoma TCGA data.

Earlier work had already suggested that TCF19 is a putative transactivation factor expressing at the late G1/S boundary in dividing cells (Ku *et al*, 1991). Recently, Krautkramer *et al* (2013) reported that TCF19 is a novel islet factor necessary for proliferation and survival in the INS‐1 β‐cell line; its downregulation affected the expression of numerous cell cycle genes from the late G1 through the M phases and resulted in cells arresting at the G1/S checkpoint. Together, these reports support the results of Echevarría‐Vargas *et al* (2018), which TCF19 plays a crucial role in BET/MEK inhibitor‐mediated blockage of tumor growth in NRAS‐mutant melanoma cells by perturbing the cell cycle machinery and activating apoptotic signaling. Interestingly, because both BRD4 knockdown and MEK inhibitor downregulates the protein level of TCF19, it could be postulated that expression of TCF19 may mechanistically be regulated by BRD4 and MEK pathways (Echevarría‐Vargas *et al*, 2018). These results suggest that TCF19 may also play an important role in melanoma with NRAS mutation and immune therapy resistance and could be used as a possible biomarker.

This has potentially direct clinical relevance: While combination treatment of BRAF/MEK inhibitors and immune checkpoint inhibition have led to durable responses and improved survival in metastatic and BRAF inhibitor‐resistant melanoma, a subgroup of patients still relapses and develops therapy resistance (Gide *et al*, 2018). The study by Echevarría‐Vargas *et al* (2018) suggests a new therapeutic approach with a molecular target. It is particularly interesting that the TCF19 gene colocalizes with the MHC class I genes, suggesting that the former may relate to tumor immunology (Cheung *et al*, 2011). Indeed, NRAS mutations in melanoma are associated with a reduced presence of tumor‐infiltrating immune cells, suggesting an immunosuppressive microenvironment, which might explain the poor response to immunotherapy (Thomas *et al*, 2015). BRD4 is also involved in the regulation of the tumor‐suppressive immunosurveillance program (Tasdemir *et al*, 2016). TCF19 might participate in managing immunosuppression in NRAS‐mutant melanoma cells.

In summary, Echevarría‐Vargas *et al* (2018) have not only developed a new therapeutic strategy for treating NRAS‐mutant and immune therapy‐resistant melanoma, but also elucidated the molecular mechanism of NRAS mutation and immune therapy resistance in melanoma. Of course, their approach of specifically targeting two cell‐signaling factors downstream of NRAS will require clinical testing to demonstrate benefits for cancer patients. In any case, the function of TCF19 in NRAS‐mutant melanoma, especially in BRAF/MAPK inhibitor and immune therapy resistance, is worthy to study further to better understand the mechanisms of how TCF19 is upregulated in NRAS‐mutant melanoma and how it governs the resistance to immune therapy.
